# Scientific Insights and Technological Advances in Gluten-Free Product Development

**DOI:** 10.3390/foods12020250

**Published:** 2023-01-05

**Authors:** Maria Papageorgiou, Theodoros Varzakas

**Affiliations:** 1 Department of Food Science and Technology, International Hellenic University, P.O. Box 141, GR-57400 Thessaloniki, Greece; 2 Department of Food Science and Technology, University of the Peloponnese, GR-24100 Kalamata, Greece

This Special Issue addresses new scientific insights and technological advances in the area of gluten-free product development with the aim of controlling gluten intolerance and autoimmune diseases.

This Special Issue publishes seven research papers and four review articles. In the paper by Gasparre et al. [1], the authors focused on the nutritional qualities of gluten-free bakery products labeled ketogenic and/or low-carb and compared them to standard gluten-free products. All of the ketogenic and/or low-carb products showed lower carbohydrate and had higher protein contents (*p* < 0.05) compared to standard products, as well as higher (*p* < 0.05) fat contents. Bokic et al. [2] investigated the effect of the addition of chicory root (20–40%) and extrusion conditions (moisture content from 16.3 to 22.5%, and screw speed from 500 to 900 rpm) on the contents of bioactive compounds (inulin, sesquiterpene lactones, and polyphenols) of gluten-free rice snacks, and found an improvement in bioactive compounds and mineral contents, as well as antioxidative activities in all extrudates compared to the pure rice control sample. The paper by Rados et al. [3] aimed to develop crackers with high fiber and low fermentable oligosaccharides, disaccharides, monosaccharides, and polyols (FODMAP) content. They found that crackers made from maize and millet flour mixtures with sourdough and chia or flax seeds were rated highest for overall impression, including improvement in taste and appearance. However, according to the authors, soluble fiber content should also be taken into account as it confines undesirable descriptive texture attributes and bitter taste.

Chochkov et al. [4] explored the effect of sourdoughs on the quality traits of gluten-free dough (composed of teff, rice, corn, and sorghum flours) and GF bread. They found that sourdough-fermented doughs were softer and more elastic compared to control dough and yielded reduced baking losses. Moreover, the most pronounced positive effect on aroma, taste, and aftertaste was attributed to the *Pediococcus pentosaceus* strain.

Dos Reis Gallo et al. [5] determined the chemical composition, antioxidant activity and capacity, and the glycemic as well as insulinemic responses of gluten-free (GF) sorghum bread. They conducted a randomized clinical trial and found that brown sorghum was superior to other genotypes.

Gazikalović et al. [6] produced gluten hydrolysates through suitable combinations of partial enzymatic hydrolysis and microwave pretreatment parameters with the aim of reduced allergenicity and the preservation of technofunctional features for food applications. Microwave treatment yielded protein hydrolysates with enhanced antioxidant and functional properties.

Laignier et al. [7] developed gluten-free bread samples using different concentrations of *Amorphophallus konjac* (a perennial plant from the subtropical regions of Southeast Asia and Africa) flour. The bread samples with konjac showed a high fiber content and lower levels of carbohydrates, hence lower calories.

The review papers deal with the use of additives to replace gluten and ensure the stability and elasticity of the dough, hence improving the nutritional quality and sensory properties of gluten-free bread [8]. The application of hydrocolloids in GF bread and pasta, affecting dough rheology, bread hardness, specific volume, staling, and the glycemic index, is discussed in [9], whereas plant-based gluten-free proteins as well as high-protein sources of animal origin, sea-microorganism- and insect-based proteins, are illustrated in [10]. Finally, sourdough biotechnology based on an ecosystem of lactic acid bacteria (LAB) and yeasts to facilitate gluten-free products is described in [11].
